# Effects of Ketogenic Diets on Cardiovascular Risk Factors: Evidence from Animal and Human Studies

**DOI:** 10.3390/nu9050517

**Published:** 2017-05-19

**Authors:** Christophe Kosinski, François R. Jornayvaz

**Affiliations:** 1 Service of Endocrinology, Diabetes and Metabolism, Lausanne University Hospital (CHUV), Avenue de la Sallaz 8, 1011 Lausanne, Switzerland; christophe.kosinski@chuv.ch; 2 Service of Endocrinology, Diabetes, Hypertension and Nutrition, Geneva University Hospitals, Rue Gabrielle-Perret-Gentil 4, 1205 Geneva, Switzerland

**Keywords:** ketogenic diets, obesity, NAFLD, fibroblast growth factor (FGF21), insulin resistance, type 2 diabetes, cardiovascular risk factors

## Abstract

The treatment of obesity and cardiovascular diseases is one of the most difficult and important challenges nowadays. Weight loss is frequently offered as a therapy and is aimed at improving some of the components of the metabolic syndrome. Among various diets, ketogenic diets, which are very low in carbohydrates and usually high in fats and/or proteins, have gained in popularity. Results regarding the impact of such diets on cardiovascular risk factors are controversial, both in animals and humans, but some improvements notably in obesity and type 2 diabetes have been described. Unfortunately, these effects seem to be limited in time. Moreover, these diets are not totally safe and can be associated with some adverse events. Notably, in rodents, development of nonalcoholic fatty liver disease (NAFLD) and insulin resistance have been described. The aim of this review is to discuss the role of ketogenic diets on different cardiovascular risk factors in both animals and humans based on available evidence.

## 1. Introduction

As a consequence of the rising obesity prevalence in industrialized countries, the incidence of cardiovascular diseases also increases [1]. Obesity is also a major risk factor for insulin resistance and type 2 diabetes [2]. This state of insulin resistance is frequently associated with ectopic lipid accumulation, notably in the liver and skeletal muscle. This can lead to the development of nonalcoholic fatty liver disease (NAFLD), which is an independent predictor of cardiovascular disease [3,4]. NAFLD is defined as steatosis which is not due to excess consumption of alcohol, viral or autoimmune causes, and iron overload [5,6]. No evidence-based pharmacological treatment for NAFLD exists so far. NAFLD is an important risk factor for the development of insulin resistance and type 2 diabetes, which may be associated with other cardiovascular risk factors such as dyslipidemia and high blood pressure. As NAFLD is present in almost 90% of obese patients [7], weight loss represents one of the pillars of the treatments among others, such as physical activity.

In the literature, diets rich in carbohydrates, and notably rich in refined sugars and fructose, are associated with the metabolic syndrome [8,9]. Therefore, to lose weight, different diets have been suggested. Among them, carbohydrate restriction has been proposed to be the single most effective intervention for reducing all features of the metabolic syndrome [10,11,12]. Since the publication of Atkins’s book in the early 1970s [13], low-carbohydrate diets have become increasingly popular, particularly ketogenic diets (KD). These diets are known for being very low in carbohydrates, but usually high in fats and/or proteins. In practice, KD are characterized by a reduction in carbohydrates (usually less than 50 g/day) and a relative increase in the proportions of proteins and fats [14]. Some variations exist, like very-low-carbohydrate KD, which are even more restrictive, with less than 30 g/day (Table 1).

After a few days of such diets, glucose reserves (i.e., glycogen stored in liver and skeletal muscle) become insufficient to provide body energy needs. This leads to the production of ketone bodies by the liver, which will be used as an alternative energy source notably by the central nervous system [18].

KD are known to be efficient in the treatment of seizures and can be used as an alternative treatment [19], but this aspect will not be discussed in this review. Nevertheless, for about forty years, the potential use of KD has also been investigated in the prevention and treatment of cardiovascular risk factors. The aim of this review is to discuss the available evidence in animal and human studies, and the role of KD on different cardiovascular risk factors, namely obesity, NAFLD, insulin resistance and type 2 diabetes, dyslipidemia and high blood pressure. To our knowledge, this is the first review comparing the effects of KD on cardiovascular risk factors in animals and humans.

## 2. Method

This article is neither a systematic review nor a meta-analysis. We searched Medline (PubMed) for trials in animals and humans, reviews or meta-analyses, using the query “ketogenic diet” + “weight loss”, “obesity”, “fibroblast growth factor (FGF21)”, “NAFLD”, “diabetes”, “insulin resistance”, “dyslipidemia” or “blood pressure”. Then, we selected the most recent papers (less than 15 years) and publications with potential practical usefulness. Finally, we only kept studies of adults, not children.

## 3. Results

### 3.1. KD and Obesity

Studies in rodents (obese or non obese) show that KD are efficient for weight loss [20,21]. Nevertheless, it is important to assess body composition changes, as it is always better to lose fat mass than lean mass. Indeed, in a study by Garbow et al., after 12 weeks KD led to a significantly lower weight gain compared to chow-fed and high-simple-carbohydrate high-fat Western diet fed mice, but lean mass was significantly reduced in KD-fed mice compared to chow-fed mice [22]. In another study, accumulation of visceral fat mass was significantly higher (at least 30%) in rats fed a KD (two compositions of KD were tested: a high-protein “Atkins-style” or a low-protein diet, both with a low-carbohydrate and high-fat content), compared with chow-fed controls, after 4 weeks of diet [23]. Finally, Jornayvaz et al. showed that KD-fed mice during 5 weeks gained significantly less weight than regular-chow fed mice. Nevertheless, KD-fed mice had an increased fat mass percentage than regular-chow fed mice, without differences in the percentage of lean body mass between diets [24].

It is also important to assess whether weight loss can be maintained. Long-term studies reveal an absence of weight loss after 22 weeks of KD in mice, despite an initial weight loss during the first week of diet [25]. Moreover, another study showed that mice fed a KD for 80 weeks initially lost weight, but after 18 weeks, their weight returned to baseline and then increased gradually [26]. Nevertheless, they gained less weight than chow-fed mice, and, with body composition analysis, the authors showed that this difference resulted from both a lower lean mass and a lower fat mass. Moreover, the survival curves were the same between the two diets. Finally, KD-fed mice also had an increased energy expenditure and a loss of the diurnal pattern of the respiratory exchange ratio, which indicated continuous use of fatty acids as an energy substrate [26]. This rise in energy expenditure was analyzed in another study which showed that KD promotes weight loss (20% of total body weight) through an increased energy expenditure and this correlated with a rise in plasma fibroblast growth factor 21 (FGF21) levels [27]. The role of FGF21 in KD will be further discussed later. In another study, compared to non-KD, rats fed a low protein (10% of total content) KD had a waste of energy in urine. [28]. Indeed, KD-fed rats had a lower urine nitrogen excretion due to a lower protein intake and a urine energy-to-nitrogen ratio almost twice as high as the other diets. 

Overall, an increase in energy expenditure in mice fed a KD compared to mice fed a chow diet could be the mechanism responsible for decreased weight gain or weight loss seen in rodents, despite a similar caloric intake [24]. As a potential component of the increased energy expenditure, other authors performed microarrays in the liver of KD-fed mice and described an increased expression of genes involved in fatty acid oxidation and a reduced expression in genes involved in lipid synthesis [20].

A recent study analyzed the effects of KD on exercising rats and sedentary rats [29]. Compared to other diets (Western diet, standard chow), after 6 weeks, sedentary KD-fed rats had an approximately 25% lower body mass, a lower size of adipocytes from omental adipose tissue, 80% lower levels of serum insulin, 50% lower levels of glucose, 55% lower levels of triglycerides and 20% lower levels of total cholesterol. Activity did not confer a benefit, as KD-fed exercising rats did not show better results than the sedentary rats. Nevertheless, exercising rats had 40% lower serum β-hydroxybutyrate levels than sedentary rats, independent of diet, while they had more favorable adipose tissue characteristics. These results suggest that body fat regulation (e.g., reduced adipose tissue mass and cell size) under a KD (with or without exercise) could rather be due to lower insulin levels. Therefore, increased serum ketones may have a smaller role.

In humans, KD are known to be an effective weight-loss therapy [30,31,32,33] (in average up to 5% of body weight at 6 months), but the mechanisms are not clearly established. Some authors suggest that it results simply from reduced caloric intake and an increased satiety effect of proteins [34]. Other studies suggest a metabolic effect of KD: possibly, the use of energy from proteins in KD is an expensive process and therefore increases weight loss [35,36,37]. Also, there is the suggestion that gluconeogenesis, which is increased with carbohydrate restriction, is energy demanding [35,38]. Another hypothesis of KD-induced weight loss is decreased appetite induced by ketosis [39]. Some authors also suggest digestive metabolic changes: with ketogenic very-low energy diets, ghrelin levels and subjective appetite (usually increased in a hypocaloric diet) were reduced when patients were in a ketotic state [39]. Surprisingly, leptin levels were lower under ketosis. A study in 132 severely obese patients (mean body mass index (BMI) 43 kg/m^2^) with a high prevalence of type 2 diabetes or metabolic syndrome showed that participants using a low-carbohydrate diet lost more weight than those using other diets, suggesting a greater reduction in overall caloric intake, rather than a direct effect of macronutrient composition [32].

As discussed in rodents, the problem is that a lot of studies are of short duration. For example, a small study [40] of 17 obese men, randomized to two different high-protein diets (one low-carbohydrate, “ketogenic”; one medium-carbohydrate, “non-ketogenic”) with a cross-over design, eating ad libitum during 4 weeks each, revealed that KD reduced hunger and was associated with a lower food intake. Weight loss was also significantly greater with the ketogenic diet than with the non-ketogenic diet, and weight loss was equally comprised of fat mass and fat-free mass. Only 35% of the difference in total weight loss between the two diets was due to water depletion; the remainder was attributed to fat mass and lean mass loss [40]. In a bigger study of 311 participants [41], a very low-carbohydrate KD followed by a period of slow re-insertion of a Mediterranean diet and alimentary education was associated with an overall improvement (mean total body weight loss of 14 ± 10 kg, BMI 5 ± 3 kg/m^2^, waist circumference 13 ± 7 cm) at 1–4 months, which remained stable after 1 year [41]. The limitations were the composition of the diet (low-carbohydrate, but also low-fat) and the fact that it was an observational study. Other authors [42] compared different diets in the same group of patients eating alternatively a KD (two different periods of 20 days), a low-carbohydrate non-KD (two different periods of 20 days), and a normal Mediterranean diet (4 months during the rotation of the two other diets, then 6 months) during 1 year. Significant weight loss and reduction of body fat percentages were observed only during ketogenic periods compared to the two other diets. Moreover, if the patients were compliant to the prescribed Mediterranean diet (which was relatively strict: 1800 kcal/day) during the maintenance period, no weight regain was observed at 12 months [42]. In an older study in obese non-diabetic participants, a low-carbohydrate diet led to a greater weight loss after 6 months, but no significant difference at 1 year [33]. The authors suggested that weight loss was probably due to a greater energy deficit, but the mechanisms remain unknown, and no relation between weight loss and ketosis was found at any time during the study. Finally, in a study with obese type 2 diabetes patients [43], the authors compared a low-carbohydrate high unsaturated fat diet to a high-carbohydrate low-fat diet, in adjunction with structured exercise: weight loss was similar in both groups (−9.1%), but a trend toward regaining more weight was observed in the low-carbohydrate diet group at 52 weeks. Weight loss is usually related to a high intensity of lifestyle interventions. Thus, it would be interesting to know the evolution on the next months/years of follow-up, and if the weight loss would be maintained without structured exercise. Exercise has an impact on body weight, but also on body composition as described in a study that analyzed the association of KD or regular diet in combination with exercise (resistance training) in overweight women [44]. The KD group lost fat mass without experiencing a significant alteration in lean mass, while the other group gained lean mass without a significant change in fat mass [44].

To summarize, in rodents and humans, KD seem to have a benefit on weight loss, notably by increased energy expenditure in animals and decreasing food intake in humans. In humans, weight loss affects both fat mass and lean mass. Greater weight loss is also associated with structured professional support, which may be difficult and expensive to maintain over time. Long-term studies are nevertheless needed to assess the evolution of weight loss. 

### 3.2. KD and NAFLD

In mice, KD induce hepatic inflammation and lipid accumulation [22], while inflammation is reduced in white adipose tissue [27]. KD also lead to hepatic steatosis in both short-term [24,25] and long-term feeding in mice [26]. Biological markers such as aspartate aminotransferase (AST) and alanine aminotransferase (ALT) are increased at least twofold in parallel with increased intrahepatic triglyceride content [24,25]. On the contrary, a study described that compared to other diets (Western diet, standard chow), KD-fed mice display reduced ALT levels, hepatic triglyceride accumulation and markers of liver inflammation [29]. The authors explained that these results, different from previous studies, could be due to the protein content of the diet (they used a KD with 20% protein, versus <10% in other studies) and differences in animal models (rat in this study, versus mice in others). Thus, this shows the importance of the diet’s composition. Another study reported an increase in hepatic triglyceride content in KD-fed mice after 12 weeks, and this correlated with an elevation in ALT levels, suggesting that chronic KD feeding causes an injury pattern similar to a NAFLD phenotype [22]. Another interesting marker of NAFLD is FGF21. In humans, FGF21 levels are increased in NAFLD and correlate with hepatic triglyceride content [45,46,47]. Nevertheless, the role of FGF21 in NAFLD induced by a KD has mostly been studied in rodents [26,27,48,49]. Notably, a study in mice showed that FGF21 plasma levels and liver expression are increased by 5 weeks of KD feeding, and this was accompanied by an increased hepatic fat content as revealed by increased hepatic triglycerides, diacylglycerols and ceramides levels [24]. Moreover, KD-fed mice developed hepatic insulin resistance and this was due to increased hepatic diacylglycerol content, as diacylglycerols are known to activate protein kinase Cε [50]. In this study [24], the authors suggested that increased plasma FGF21 levels and hepatic expression in KD-fed mice was probably secondary to hepatic fat accumulation and may represent a compensatory mechanism to counteract hepatic insulin resistance, suggesting that FGF21 may be beneficial in reversing hepatic insulin resistance. This has been further verified in high-fat diet fed mice treated with exogenous FGF21. In a study in wild-type mice, FGF21 decreased hepatic fat content, notably hepatic diacylglycerol content, and improved hepatic insulin sensitivity [51]. The beneficial role of FGF21 on hepatic insulin sensitivity was also shown by other authors [52]. Moreover, mice lacking FGF21 gain weight, have an increased fat mass and develop glucose intolerance. Moreover, when the chow diet was changed for a KD, these mice not only gained weight and developed glucose intolerance, but also developed NAFLD [53]. Finally, another study revealed that FGF21 knock-out mice fed a KD develop NAFLD and severe hepatic insulin resistance as assessed by the gold-standard technique, the hyperinsulinemic-euglycemic clamp [54]. Finally, FGF21 has been shown to act as an endocrine signal of protein restriction [55]. In summary, these studies suggest an important role of FGF21 in the pathophysiology of NAFLD.

In humans, liver fat content was shown to be increased during an isocaloric high-fat low-carbohydrate diet [56]. This result should be analyzed with caution, as with 31% of carbohydrates the diet is not a “real” KD. Caloric restriction also had an impact: compared to the high-carbohydrate (“standard”) hypocaloric diet, reduction of liver fat content was significantly higher with the hypocaloric low-carbohydrate diet [57,58]. This effect was limited in time, with no significant difference at 11 weeks [57]. KD have also been associated with a higher decrease in liver volume compared with a standard hypocaloric diet, probably due to the depletion of liver glycogen [59]. Finally, the response to KD may be influenced by genetic predisposition to NAFLD, as shown by two studies with a better response to KD for patients with variants of the PNPLA3 gene [60,61]. When fed a KD, subjects with PNPLA3 variants had a lower liver fat content than controls.

To summarize, the effect of KD on the liver was mostly investigated in rodents. The results are rather negative, with induction of hepatic inflammation and NAFLD, but these findings have not been reported in humans. An alteration of FGF21 expression could be a potential cause or consequence. More studies are warranted in humans to assess whether KD could induce or improve NAFLD.

### 3.3. KD and Insulin Resistance / Type 2 Diabetes

Obesity is often associated with the development of NAFLD, insulin resistance and type 2 diabetes. Notably, as already discussed, KD can lead to decreased weight gain in mice but with concomitant development of NAFLD and associated hepatic insulin resistance [24]. In the latter study, mice fed a KD for 5 weeks developed whole body insulin resistance despite reduced basal plasma glucose and insulin levels. In this case, the use of indices of insulin sensitivity, such as the homeostatic model assessment for insulin resistance (HOMA-IR) and quantitative insulin-sensitivity check index (QUICKI) indices, would lead to the conclusion that insulin sensitivity is improved in KD fed mice [62]. However, using the hyperinsulinemic-euglycemic clamp, glucose infusion rates were 47% lower in KD-fed mice than in chow-fed mice, demonstrating whole body insulin resistance in KD-fed mice [24]. KD-fed mice also had an impaired insulin ability to suppress endogenous glucose production confirming insulin resistance at the level of the liver. In this case, KD induced severe hepatic insulin resistance in mice despite lower body weight gain, and this was attributed to an increased hepatic diacylglycerol content. Finally, insulin resistance was also attributed to a decreased insulin-stimulated whole body glucose disposal, which was notably due to decreased glucose uptake in brown adipose tissue and the heart [24].

In another study, mice fed a KD during 12 weeks remained euglycemic, but had reduced mean serum insulin levels and HOMA-IR indices, and exhibited glucose intolerance as assessed by intra-peritoneal glucose tolerance tests [22]. However, despite mild hepatic steatosis, systemic response to insulin was preserved, unlike in other studies. The authors explained this discrepancy by a relatively reduced lean body mass in KD-fed mice, resulting in higher insulin dose in insulin-tolerance tests. Also, hepatic insulin resistance may confer a smaller contribution to overall glucose homeostasis than peripheral glucose disposal [22]. In studies using rats, KD also induced glucose intolerance and insulin resistance [23,63], despite reduced glucose and insulin levels [23]. In the latter study, the authors showed that these effects were not due to energy overconsumption. Moreover, as KD-fed rats had a significant accumulation of visceral fat, the effects on glucose homeostasis were not dependent upon visceral fat mass. In the same study, short term KD feeding in rats was also associated with decreased β-cell mass, but this effect could be due to a lower lean body mass of KD fed rats [23]. Nevertheless, these findings were corroborated by another study where long term KD feeding in mice led to glucose intolerance that was associated with insufficient insulin secretion from β-cells, potentially due to a decrease in β-cell mass [25]. Interestingly, after only 6 days of KD feeding, mice showed impairments in glucose tolerance and insulin sensitivity, and this was attributed to a possible adaptation to maintain blood glucose levels against insufficient amounts of carbohydrates [48]. In this case, insulin signaling was impaired only in white adipose tissue, but not in liver and muscle. The authors suggested that this impairment in white adipose tissue could not be the only culprit for whole body glucose intolerance in KD fed mice. Indeed, a low-carbohydrate diet might account for an impaired nutritional state compared to a chow diet. These results were explained by a lower lean mass and a proportionally higher insulin dose in insulin tolerance tests. The possible role of KD in inducing insulin resistance is nevertheless controversial. Indeed, several authors reported that long term KD fed mice had normal glucose tolerance, lower baseline insulin levels and improved insulin sensitivity [26,29].

In humans, the effect of KD on glucose homeostasis is more controversial, and notably depends on the presence of type 2 diabetes or not at baseline. A study showed that a high-fat, low-carbohydrate intake reduces the ability of insulin to suppress endogenous glucose production in healthy men, by using the gold standard method, the hyperinsulinemic-euglycemic clamp [64]. The limitation is the small number of participants (only 6). Other studies described the opposite: with a KD, obese non-diabetic participants had a significant lower HOMA-IR and fasting glucose after a KD than at baseline [40], and an improvement in insulin sensitivity using the QUICKI [32]. Finally, another trial also described a better insulin sensitivity by consuming a KD [33], but this improvement does not seem to be permanent. Indeed, Forster et al. found a significant improvement in insulin sensitivity at 6 months, but not at 1 year [33]. This reduction in insulin levels could be explained by the satietogenic effect of this diet [65]. 

In type 2 diabetic patients, KD could be an interesting approach, as most patients have glycemic variability due to food carbohydrate content. A lot of studies have been designed around this purpose. KD are frequently associated with a diminution of blood glucose levels (up to 0.5 mmol/L) [41,66,67,68], glycosylated hemoglobin (HbA1c (up to 0.3%) [41,66,68,69], glycemic variability [70] and improvement in insulin sensitivity [32,68], sometimes without weight loss [67]. Low-carbohydrate diets can lead to a reduction in medications in type 2 diabetic patients [69,70,71,72,73,74]. An important aspect is to assess the long-term effect of a KD on these outcomes. In studies in obese type 2 diabetic patients fed a KD, a significant improvement in fasting glucose levels was seen after 12 weeks and continued after 56 weeks [75]. Nevertheless, in another study, a short-term decrease in HbA1c was observed at 6 months, but was not sustained at 24 months [76]. The latter study is probably more relevant to daily clinical practice because it was a low-intensity intervention. A recent study in obese type 2 diabetic patients compared a hypocaloric high-unsaturated/low-saturated fat very-low-carbohydrate diet to a high-carbohydrate low-fat diet [43]. After 52 weeks, the low-carbohydrate diet group showed a decrease in glycemic variability two times greater that the low-fat diet group, which indicates a greater diurnal blood glucose stability. However, improvement in HbA1c was similar in both diets (−1%). Nevertheless, the low-carbohydrate diet led to a greater reduction in antidiabetic medications, which could overall be helpful to optimize glycemic control [43]. Still, long-term effects of such dietary changes need to be evaluated.

In a prospective cohort of non-diabetic men, health professionals younger than 65 years old followed during 20 years, a low-carbohydrate diet high in animal proteins and fats was associated with a twofold increased risk of type 2 diabetes [77]. On the other hand, a low-carbohydrate diet high in vegetal proteins and fats was associated with a decreased risk of type 2 diabetes [77]. These findings suggest that a low-carbohydrate diet should contain proteins and fats from foods other than red and processed meat. However, these results were found from non-ketogenic diets. Therefore, further studies with “vegetal versus animal protein and fat content” KD are necessary to assess if these assumptions are validated. In another trial [32], a greater decrease of mean fasting glucose levels (−9%) was observed in all subjects of the low-carbohydrate group, but was significant only in diabetic patients (−15%), with more reduction of oral hypoglycemic agents or insulin in the low-carbohydrate group. Interestingly, in a study in obese non diabetic patients, after 8 weeks, a very-low-energy KD led to a rise in postprandial glucose levels, but not in fasting glucose levels [39]. The authors postulated that KD reduces insulin ability to suppress endogenous glucose production and impairs insulin-stimulated glucose oxidation, suggesting that there may be a different effect of ketosis on glucose homeostasis between diabetic and non-diabetic patients. For example, in obese type 2 diabetic patients, a strong inverse correlation between circulating ketones and hepatic glucose output has been described [78], suggesting that higher levels of ketones are associated with more favorable effects on glycemic control in these subjects.

An interesting study using biology systems approaches found a strong relationship between the insulin resistance pathway and the ketosis main pathway, providing a possible explanation for the improvement in glucose homeostasis found in clinical trials using low-carbohydrate diets. Notably, maps analyses suggest a direct implication of glucose transporters and inflammatory processes [79].

In summary, in rodents, KD mostly induces insulin resistance and glucose intolerance, while in type 2 diabetic humans KD is associated with a better control in glucose homeostasis and a reduction in antidiabetic medications. Nevertheless, these improvements seem to be limited in time. Further studies should evaluate if a higher weight loss correlates with a better glucose control or with higher ketones levels. 

### 3.4. KD and Dyslipidemia 

Dyslipidemia is a well-known risk factor for cardiovascular diseases. As KD are usually high in fats, it is necessary to assess their potential effect on the lipid profile.

In rodents, short term (14 days) studies showed no change in fatty acids and triglycerides levels in mice fed a KD [48]. The duration and the composition of KD feeding are very important. In a study of 4 weeks, Bielohuby et al. compared the effects on rats of chow diet and two different KD: one with 78.7% fat, 19.1% protein, 2.2% carbohydrates, and the other with 92.8% fat, 5.5% protein, 1.7% carbohydrates. The very high-fat KD-fed group had a reduction in high-density lipoproteins (HDL) cholesterol levels and higher triglycerides levels. No significant difference in total cholesterol levels was found between the three groups [23]. It should be noticed that the authors did not mention the effect on low-density lipoproteins (LDL) cholesterol levels. In another study, Jornayvaz et al. showed that mice fed a KD during 5 weeks had similar levels of LDL cholesterol than mice fed a chow diet [24]. In longer term (28–80 weeks) studies, KD fed mice displayed a twofold increase in plasma total cholesterol and triglyceride levels [25,26]. On the opposite, a recent study reported that mice fed a KD during 6 weeks had lower total cholesterol and triglycerides levels than with other diets [29]. The authors suggested that there was a KD-induced reduction in insulin levels, which further decreased liver fatty acids and cholesterol biosynthesis pathways [20,29]. Overall, most studies in animals used KD rich in saturated fats, which may have detrimental effects on the lipid profile compared to KD rich in unsaturated fats, and therefore limits definitive conclusions on the role of KD on dyslipidemia.

In humans, KD have been associated with significant reductions in total cholesterol [75], increases in HDL cholesterol levels [33,70,75,80,81,82,83], decreases in triglycerides levels [32,33,70,75,82,83] and reductions in LDL cholesterol levels [75]. These results were obtained in non epileptic obese participants with [32,70,75,83] or without [33,82] at least one risk factor of the metabolic syndrome, but also in healthy normal weight participants [80]. This is of importance as the effects of KD on the lipid profile may differ in epileptic subjects [84]. KD have also been associated with an increase in size and volume of LDL cholesterol particles, which is considered to reduce cardiovascular risk by decreasing atherogenicity [81]. Nevertheless, several studies showed an increase in LDL cholesterol levels [82,83,85,86], but not significantly in the trial by Westman et al. [85]; in these cases, KD were mostly composed of saturated fats. In other studies, total and LDL cholesterol were significantly more reduced with a high-protein medium-carbohydrate diet than with a KD [40]. Another study [33] reported no significant difference in total and LDL cholesterol levels after 12 months of a KD compared to a conventional diet, except at 3 months, where LDL cholesterol levels were lower in the conventional diet group. The absence of improvement suggests that weight loss with a low-carbohydrate diet is not associated with a decrease in LDL cholesterol usually observed with moderate weight loss [33]. Interestingly, the effect of a KD on lipid profile may be associated with ethnicity: in a study, white subjects lost more weight and had a bigger decrease in triglycerides levels than black subjects [32]. However, in this study, there was no significant change in total cholesterol, HDL cholesterol and LDL cholesterol levels.

As discussed for other cardiovascular risk factors, the composition of the diet used is very important. For example, in a study that reported a benefit of a KD on triglycerides and HDL cholesterol levels [70], the authors decided to use a low-carbohydrate diet rich in unsaturated but low in saturated fatty acids, which may greatly influence the lipid profile, but also the development of other metabolic complications such as NAFLD, insulin resistance and type 2 diabetes, as discussed earlier.

The impact of KD on the lipid profile differs between rodents and humans. In rodents, KD seem to be associated with worsened levels of total, HDL and LDL cholesterol, and triglycerides. In humans, the opposite is reported. These differences are mostly explained by differences in the composition of the diets, which are usually higher in total fat, but also in saturated fat in animal studies. Both in rodents and humans, comparison between saturated fat and unsaturated fat KD in long-term studies would be necessary. Later in this review, we will discuss whether saturated fat diets are as harmful as once thought. 

### 3.5. KD and Blood Pressure

Studies reporting a potential effect of KD on blood pressure are scarce. We could not find studies in animals. In humans, KD are usually associated with a slight but not significant reduction in systolic and/or diastolic blood pressure [32]. Moreover, no change in antihypertensive therapy was observed [32,33]. Nevertheless, a study described an improvement in both systolic and diastolic blood pressure in obese participants when fed a KD during 48 weeks compared to a low-fat diet plus orlistat [69]. Weight loss was not a confounding factor as weight loss was similar in both arms. Finally, a reduction in systolic blood pressure was found in a study using a KD, but only after 3 months, without change after 1 year of observation [41].

Overall, there is a clear lack of conclusive data on the potential beneficial effect of KD on arterial blood pressure and further studies are therefore needed.

## 4. Discussion 

**Figure 1:**
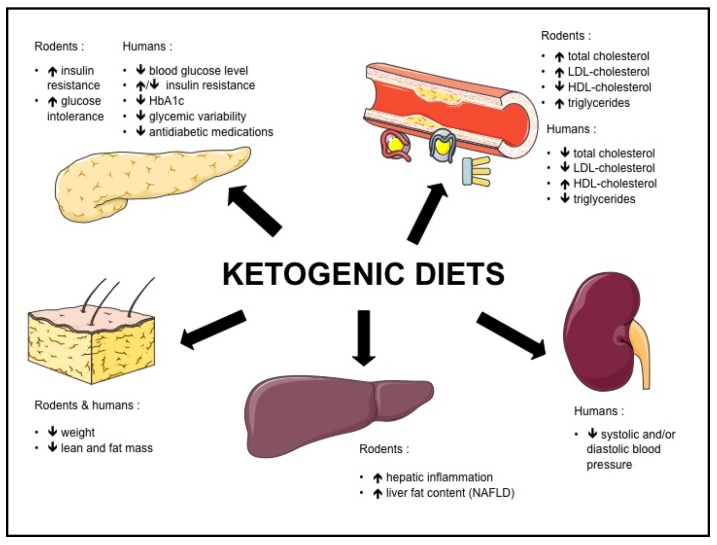
Effects of ketogenic diets in rodents and humans.

**Figure 2:**
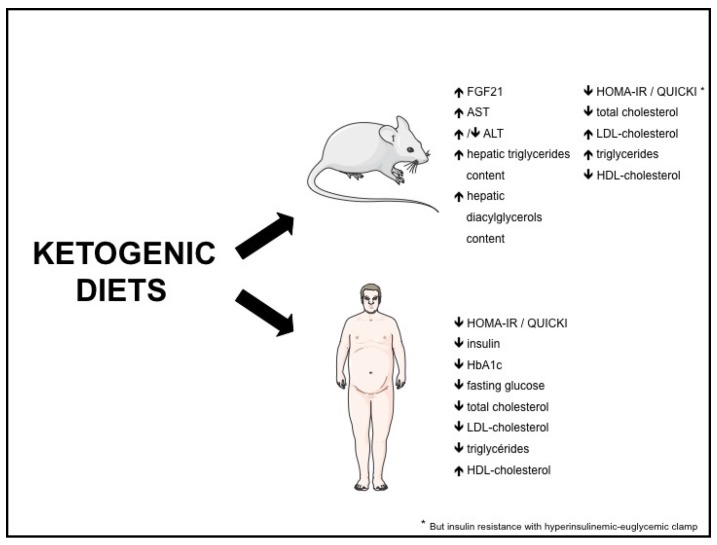
Effects of ketogenic diets on biomolecular markers. FGF21: fibroblast growth factor-21; ALT: alanine aminotransferase; AST: aspartate aminotransferase; QUICKI: quantitative insulin-sensitivity check index; HOMA-IR: homeostasis model assessment of insulin resistance.

Both in rodents and humans, controversies remain regarding the effect of KD on metabolic risk factors such as NAFLD, insulin resistance, type 2 diabetes and dyslipidemia. The potential beneficial and adverse effects of KD are summarized in Figure 1, and the effects of KD on different biomolecular markers in Figure 2. 

Overall, KD composition greatly differs between studies. KD used in rodents are usually not similar to KD used in humans (almost no carbohydrates and low protein content in rodent KD). Moreover, low-carbohydrate diets can be different in macronutrient composition, i.e., high-fat versus high-protein content, which may account for some of the differences between the studies. Moreover, as previously mentioned, fat composition can substantially differ between studies, some using KD rich in unsaturated fatty acids and others rich in saturated fatty acids. Nevertheless, a recent systematic review and meta-analysis revealed that saturated fat intake was not associated with all-cause and cardiovascular mortality, coronary heart disease, ischemic stroke or type 2 diabetes, but with heterogenous evidence [87]. The authors conclude that trans fats were associated with all-cause and cardiovascular mortality and also with coronary heart disease. Moreover, it is well known that different ratios of some unsaturated and saturated fatty acids in diet compositions can alter metabolic parameters such as insulin sensitivity [9]. Therefore, it is a real challenge for physicians to advise patients with different metabolic diseases about the best diet composition to use. If a KD has to be prescribed, maybe it could be better to favor a vegetable-based KD, as vegetable-based low-carbohydrate diets have been correlated with a decrease in all-cause and cardiovascular-related mortality [88]. In the latter study, two US cohorts (121,700 females, 51,529 males) were followed during 26 and 20 years, respectively. Both in men and women, animal-based low-carbohydrate diets were found to be associated with higher all-cause (especially cardiovascular mortality) and cancer mortality, compared to vegetable-based low-carbohydrate diets. Nevertheless, similar studies with “real” KD need to be performed to confirm this assumption, as a low-carbohdyrate diet is not necessarily inducing ketosis and is therefore not a ketogenic diet per se. Another problem when using a KD is the long-term effect and sustainability of effects, notably due to a lack of long-term studies in metabolic diseases such as type 2 diabetes. Restrictive diets are often associated with poor long-term adherence [89]. Nevertheless, some evidence suggests that adherence to low-carbohydrate diets is better than to low-fat diets, because of the allowance to unlimited access to food as long as carbohydrates are reduced, given that proteins and fats are known to induce satiety [16].

Three meta-analyses about the effect of KD on cardiovascular risk factors were published recently [90,91,92]. Their conclusions are unanimous about general positive effects, but not unanimous about each single variable. Santos et al. concluded in 2012 that low-carbohydrate diets lead to a significant decrease in body weight, BMI, abdominal circumference, both systolic and diastolic blood pressure, triglycerides levels, fasting plasma glucose and HbA1c, an increase in HDL cholesterol levels, and no change in LDL cholesterol levels. As we mentioned before, the authors suggested a possible duration effect, specifically for body weight and blood pressure, where benefits seem to decrease over time [90]. Bezerra Bueno et al. compared very-low carbohydrate diets to low-fat diets and their effects after a follow-up of at least 12 months. Very-low carbohydrate diets confer a greater weight loss, reduction in triglycerides and diastolic blood pressure, and increase in HDL and LDL cholesterol levels. There was however no difference in systolic blood pressure. There was no significant difference between diets for fasting blood glucose and insulin levels, and HbA1c. It is interesting to note that in the studies (only 4) with 24 months of follow-up, only the change in HDL cholesterol levels remained significant [91]. The latest meta-analysis on KD by Naude et al. included 19 randomized controlled trials (RCT) and revealed that there is probably little or no difference in changes in weight or cardiovascular risk factors when comparing low-carbohydrate diets to isoenergetic diets (both showed weight loss) after two years of follow-up. These results were found in overweight and obese patients, with or without type 2 diabetes. This meta-analysis showed that strict adherence failed and declined with follow-up in most trials [92].

It is also important to keep in mind that KD could have some adverse side effects when chronically used. Indeed, studies in children using KD to treat epilepsy and other neurological disorders show an increase in kidney stones, osteoporosis, hyperlipidemia and impaired growth [93,94]. While several authors found that a low-carbohydrate high-protein diet was not associated with higher mortality after 12 years of follow-up [95], others described a weak statistically significant higher mortality rate after 10 years [96]. However, they did not evaluate sources of proteins and fats.

Finally, it should be mentioned that it is difficult to really know in studies if low-carbohydrate diets were “real” KD. Indeed, most of the time there is no report about a potential induction of ketosis, by for example reporting measurements of plasma ketone bodies.

## 5. Conclusions

Based on the available literature, KD may be associated with some improvements in some cardiovascular risk factors, such as obesity, type 2 diabetes and HDL cholesterol levels, but these effects are usually limited in time. As KD are often rich in fats, some negative effects could happen. Mainly in rodents, developments of NAFLD and insulin resistance were described. In humans, insulin resistance is also a potential negative effect, but some studies have shown improvements in insulin sensitivity. Nevertheless, many subjects contemplating such diets are overweight or obese at baseline, and even a moderate weight loss could be metabolically beneficial for them. However, it is mandatory to maintain body weight after weight loss, which is usually a major problem. More studies are therefore warranted to better assess the effects of long term use of KD on metabolic diseases and cardiovascular risk factors, but also to better define which dietary macronutrient composition is optimal.

## Figures and Tables

**Table 1:** Standard composition of ketogenic diets in adults * (calculated for a 2000 kcal diet/day).

Classical KD	Defined as <130 g carbohydrate per day or <26% of caloric intake by the American Diabetes Association
Modified Atkins Diet	65% caloric intake from fat, 30% protein, 6% carbohydrates
Very low-carbohydrate KD	Carbohydrates < 30 g/day

KD, Ketogenic Diet. * Adapted from Kossoff et al. [15], Feinman et al. [16], Accurso et al. [17].
